# Adaptive Backstepping Design of a Microgyroscope

**DOI:** 10.3390/mi9070338

**Published:** 2018-07-03

**Authors:** Yunmei Fang, Juntao Fei, Yuzheng Yang

**Affiliations:** College of Electrical and Mechanical Engineering, Hohai University, Changzhou 213022, China; yunmeif@163.com (Y.F.); smithcopy@163.com (Y.Y.)

**Keywords:** adaptive control, backstepping approach, tracking performance, microgyroscope

## Abstract

This paper presents a novel algorithm for the design and analysis of an adaptive backstepping controller (ABC) for a microgyroscope. Firstly, Lagrange–Maxwell electromechanical equations are established to derive the dynamic model of a *z*-axis microgyroscope. Secondly, a nonlinear controller as a backstepping design approach is introduced and deployed in order to drive the trajectory tracking errors to converge to zero with asymptotic stability. Meanwhile, an adaptive estimator is developed and implemented with the backstepping controller to update the value of the parameter estimates in the Lyapunov framework in real-time. In addition, the unknown system parameters including the angular velocity may be estimated online if the persistent excitation (PE) requirement is met. A robust compensator is incorporated in the adaptive backstepping algorithm to suppress the parameter variations and external disturbances. Finally, simulation studies are conducted to prove the validity of the proposed ABC scheme with guaranteed asymptotic stability and excellent tracking performance, as well as consistent parameter estimates in the presence of model uncertainties and disturbances.

## 1. Introduction

As primary information sensors, microgyroscopes have a large potential for several types of applications in navigation, control, and guidance systems. Fabrication imperfections in microgyroscopes always generate some coupling between oscillation modes. Meanwhile, the performance of the microgyroscope is subject to quadrature errors, time-varying parameters, and external disturbances. Nevertheless, recent applications require sensors with improved performance. The incorporation of advanced control systems into their existing dynamics seems to be an effective way to improve the microgyroscope performance.

During the past decades, many researchers have spent great deal of effort in the design of microgyroscope structures and control systems [1,2,3,4,5,6,7,8,9,10,11,12,13,14,15,16,17]. The conventional controller for a microgyroscope is to force the drive mode into a known oscillatory motion and then detect the Coriolis effect coupling along the orthogonal sense mode, which provides the information about the applied angular velocity. However, the conventional controllers are immanently sensitive to some typical types of fabrication imperfections, such as the cross-damping term, which produces zero-rate output. To solve these problems, advanced control schemes such as adaptive controller [2,3,4,5], sliding mode controller [6], compound robust controller [7], adaptive neural controller [8,9,10], and adaptive fuzzy controller [11,12,13] have been applied to microgyroscopes. A mode-matched force-rebalance control for a microgyroscope was investigated in [14]. Adaptive dynamic surface control for a triaxial microgyroscope with nonlinear inputs was developed in [15]. Flatness-based adaptive fuzzy control of an electrostatically actuated micro-electro-mechanical system (MEMS) and self-adaptive nonlinear stops for mechanical shock protection of MEMS were discussed in [16,17], respectively.

A backstepping controller [18] that can achieve the goals of tracking and stabilization is a recursive design procedure based on a Lyapunov framework, breaking a full system design into a sequence of lower-order systems. Nevertheless, compared with sliding mode control, the backstepping algorithm has two merits: the first is that it can relax the matching condition for a class of systems which can satisfy the strict feedback form; the second is that it can refrain from cancellation of the useful nonlinearities existing in the nonlinear system. The fundamental rule of backstepping is to recursively design a controller and step back out of the subsystem progressively, guaranteeing stability at each step, until reaching the final external control step. In [19,20], adaptive backstepping controllers were deployed for an air-breathing hypersonic vehicle and a fuel cell/boost converter system. A backstepping controller was applied to a linear 2 × 2 hyperbolic system in [21]. Adaptive intelligent control with backstepping design for dynamic systems were developed in [22,23,24,25,26]. Adaptive command-filtered backstepping control of robot arms with compliant actuators was introduced in [27]. However, so far, an adaptive backstepping controller has not been deployed to a microgyroscope. Based on our preliminary work in [28], our work will explore an adaptive backstepping scheme with a parameter estimator for a microgyroscope. Compared with existing works, the main contributions of the proposed backstepping approach are emphasized as:

(1) Backstepping is a nonlinear control approach based on Lyapunov stability theorem by means of recursion process. Backstepping design is a powerful tool for dynamic systems with pure or strict feedback forms. A major advantage of backstepping is that it has the flexibility to avoid cancellations of useful nonlinearities and achieve regulation and tracking properties. However, the vibratory microgyroscope is neither of these two forms. Therefore, the microgyroscope motion equations should be transformed into a cascade-like system to be suitable for the backstepping approach.
(2) An adaptive control strategy is deployed in the backstepping procedure to deal with parameter uncertainties and external disturbances. The Lyapunov-based adaptive controller is obtained to guarantee the asymptotic stability of the closed-loop system and the consistent parameter estimates, including the external angular velocity if the persistent excitation (PE) condition is satisfied. In addition, a robust term is incorporated in the adaptive backstepping algorithm to suppress the lumped disturbances.

## 2. Microgyroscope Dynamics

**Figure 1:**
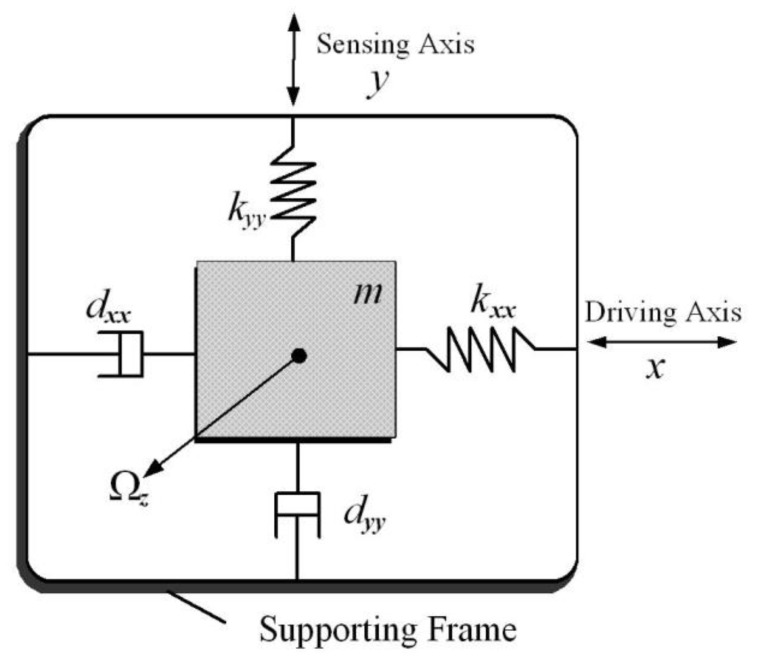
Schematic model of a *z*-axis MEMS vibratory gyroscope.

A *z*-axis vibratory microgyroscope mainly consists of three components: the sensitive element; electrostatic actuations and sensing mechanisms; and the rigid frame rotating along the rotation *z*-axis. Figure 1 shows a schematic diagram of a microgyroscope. The motion equations of the microgyroscope are developed from the Lagrange–Maxwell equation [1,2]:

(1) $$\frac{d}{dt}\left(\frac{\partial L}{\partial {\dot{x}}_{i}}\right)-\frac{\partial L}{\partial x_{i}}+\frac{\partial F}{\partial {\dot{x}}_{i}}=Q_{i},$$

where $L=E_{K}-E_{P}$ is Lagrange’s function, $E_{K}$ and $E_{P}$ are kinetic and potential energies of the sensitive element, respectively, F is the generalized damping force, $Q_{i}$ are generalized forces acting on the sensitive element, and i ranges from 1 corresponding to the number of considered degrees of freedom (2 in our system).

The motion equations can be obtained according to (1) and coordinate transformation knowledge. Assuming that the angular velocity is almost constant over a sufficiently long time interval, $\Omega_{x}\approx \Omega_{y}\approx 0$, only the component of the angular velocity $\Omega_{z}$ causes a dynamic coupling between the *x*-*y* axes. Considering fabrication imperfections, which cause extra coupling, the motion equations are obtained as:

(2) $$\left\{ \begin{array}{l} m\ddot{x}+d_{xx}\dot{x}+d_{xy}\dot{y}+k_{xx}x+k_{xy}y=u_{x}+d_{x}+2m\Omega_{z}\dot{y}\\ m\ddot{y}+d_{xy}\dot{x}+d_{yy}\dot{y}+k_{xy}x+k_{yy}y=u_{y}+d_{y}-2m\Omega_{z}\dot{x} \end{array}\right.,$$

where x and y are the coordinates regarding the gyro frame existing in Cartesian coordinates; m is the mass; $d_{xx},d_{yy},k_{xx},k_{yy}$ are called the damping and spring coefficients; $d_{xy},k_{xy}$ are called quadrature errors, which are coupled damping and spring terms, respectively; $u_{x},u_{y}$ are called control forces; and $d_{x},d_{y}$ represent bounded unknown disturbances (note that the lumped disturbances $d_{x}$ and $d_{y}$ could also contain the effects of the time-varying unknown but bounded parameter uncertainties); and $2m\Omega_{z}\dot{y},2m\Omega_{z}\dot{x}$ are the Coriolis forces used to reconstruct the information of the unknown angular velocity $\Omega_{z}$.

Dividing both sides of the motion Equation in (2) by reference mass m, reference length $q_{0}$, and natural resonance frequency $\omega_{0}^{2}$, we get the non-dimensional equation as:

(3) $$\begin{array}{l} \ddot{x}+d_{xx}\dot{x}+d_{xy}\dot{y}+\omega_{x}{}^{2}x+\omega_{xy}y=u_{x}+2\Omega_{z}\dot{y}+d_{x}\\ \ddot{y}+d_{xy}\dot{x}+d_{yy}\dot{y}+\omega_{xy}x+\omega_{y}{}^{2}y=u_{y}-2\Omega_{z}\dot{x}+d_{y} \end{array},$$

where $\frac{d_{xx}}{m\omega_{0}}\to d_{xx}$, $\frac{d_{xy}}{m\omega_{0}}\to d_{xy}$, $\frac{d_{yy}}{m\omega_{0}}\to d_{yxy}$, $\frac{\Omega_{z}}{\omega_{0}}\to \Omega_{z}$, $\sqrt{\frac{k_{xx}}{m\omega_{0}{}^{2}}}\to \omega_{x}$, $\sqrt{\frac{k_{yy}}{m\omega_{0}{}^{2}}}\to \omega_{y}$, $\frac{k_{xy}}{m\omega_{0}{}^{2}}\to \omega_{xy}$.

Equation (3) can be transformed into the vector form equation as:

(4) $$\ddot{q}+D\dot{q}+Kq=u-2\Omega \dot{q}+d,$$

where $q=\left[\begin{array}{c} x\\ y \end{array}\right]$, $u=\left[\begin{array}{c} u_{x}\\ u_{y} \end{array}\right]$, $d=\left[\begin{array}{c} d_{x}\\ d_{y} \end{array}\right]$, $D=\left[\begin{array}{cc} d_{xx} & d_{xy}\\ d_{xy} & d_{yy} \end{array}\right]$, $K=\left[\begin{array}{cc} k_{xx} & k_{xy}\\ k_{xy} & k_{yy} \end{array}\right]$, $\Omega =\left[\begin{array}{cc} 0 & -\Omega_{z}\\ \Omega_{z} & 0 \end{array}\right]$. Note that $D=D^{T},K=K^{T},\Omega =-\Omega^{T}$ and the input disturbances are assumed to be bounded by ‖d‖≤ρ, where ρ is a scalar.

Considering a system with parametric uncertainties and external disturbances, the dynamics of the microgyroscope (4) can be represented as:

(5) $$\ddot{q}+(D+2\Omega +\Delta D)\dot{q}+(K+\Delta K)q=u+d,$$

where ΔD is the unknown parameter uncertainties of D+2Ω, and ΔK is the unknown parameter uncertainties of K.

Rewriting Equation (5) as

(6) $$\ddot{q}+(D+2\Omega )\dot{q}+Kq=u+d_{f},$$

where $d_{f}=d-\Delta D\dot{q}-\Delta Kq$, representing the matched, lumped parametric uncertainties and external disturbances. 

Despite these difficulties, an adaptive backstepping control (ABC) algorithm is deployed to guarantee the tracking performance, asymptotic stability, and parameter estimations of the microgyroscope system in the following section.

## 3. Adaptive Backstepping Control Design

**Figure 2:**
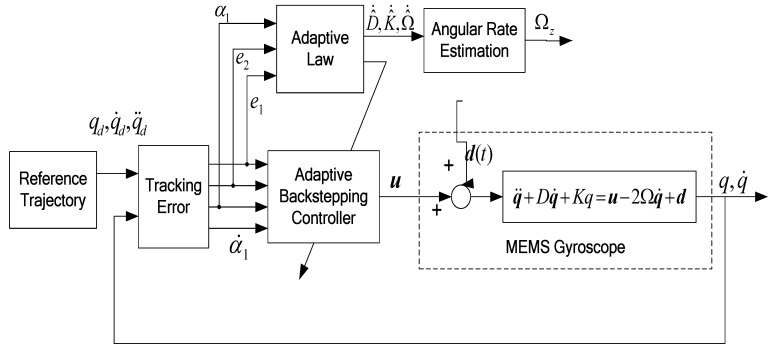
Block diagram of the proposed adaptive backstepping control of a microgyroscope.

Motivated by the research results in [18,19,20,21,22], a backstepping controller was to achieve the goals of tracking and stabilization by a recursive design procedure. We firstly show that if the parameters of the microgyroscope are known, the backstepping controller guarantees zero tracking error and asymptotic stability. Then, we will utilize an adaptive backstepping scheme to deal with the case of the unknown parameters. Figure 2 describes the block diagram of the proposed ABC approach of a microgyroscope.

As seen from Equation (3), since the coupled microgyroscope motion equation is not formulated in “strict-feedback” form, it should to be transformed into a form which could make backstepping design approach available. We define $X_{1}=q,X_{2}=\dot{q}$.

The dynamics in (3) can be transformed as the following cascade form:

(7) $$\{\begin{array}{c} {\dot{X}}_{1}=X_{2}\\ {\dot{X}}_{2}=-(D+2\Omega )X_{2}-KX_{1}+u+d_{f} \end{array}.$$

The control objective for a *z*-axis microgyroscope is to track a reference oscillation trajectory $q_{d}$ as closely as possible and make all the signals in the closed-loop system be uniformly bounded. For the microgyroscope in (5), the backstepping control design can be synthesized in two steps.

Step 1: Treat $X_{2}$ as a virtual control force and design a control law for it to make $X_{1}$ follow the reference trajectory.

Firstly, the tracking error is defined as $e_{1}=q-q_{d}=X_{1}-q_{d}$, where $q_{d}$ is the reference trajectory of q. Assume the first and second derivatives of the reference trajectory $q_{d}$ are all bounded. Considering D,K,Ω are known, we treat $X_{2}$ as a control input and design a virtual controller $\alpha_{1}$ for it such that $\lim_{t\to \infty }q=q_{d}$(i.e., $\lim_{t\to \infty }e_{1}(t)=0$). To make the tracking error $e_{1}$ converge to zero, we study the dynamics of $e_{1}$ derived by differentiating the both sides of $e_{1}=X_{1}-q_{d}$, then we obtain ${\dot{e}}_{1}=X_{2}-{\dot{q}}_{d}$.

Now that $X_{2}$ is treated as a control input, we naturally design the following simple virtual control law for $X_{2}$ to make $e_{1}$ converge to zero exponentially:

(8) $$X_{2}=\alpha_{1}\equiv -c_{1}e_{1}+{\dot{q}}_{d},$$

where $c_{1}$ is a positive definite symmetric matrix.

With virtual control law (8), the dynamics of ${\dot{e}}_{1}=X_{2}-{\dot{q}}_{d}$ become

(9) $${\dot{e}}_{1}=-c_{1}e_{1}.$$

Due to the positive property of $c_{1}$, tracking error $e_{1}$ will approach zero exponentially. Roughly speaking, $X_{1}$ rapidly approximates to $q_{d}$.

Step 2: However, $X_{2}$ is not the actual control input, but a state variable. We cannot operate $X_{2}$ directly. So, let us move on to the second line of (5), which reveals the dynamics of $X_{2}$. We design the real control force to make $X_{2}$ converge to $\alpha_{1}$.

Define $e_{2}$ as an error variable that is the deviation between $X_{2}$ and its virtual control law $\alpha_{1}$, that is, $e_{2}=X_{2}-\alpha_{1}.$

We derive the dynamics of $e_{2}$ as

(10) $$\begin{array}{ll} {\dot{e}}_{2} & ={\dot{X}}_{2}-{\dot{\alpha }}_{1}\\ & =-(D+2\Omega )(e_{2}+\alpha_{1})-K(e_{1}+q_{d})+u+d_{f}-{\dot{\alpha }}_{1}\\ & =-(D+2\Omega )e_{2}-K(e_{1}+q_{d})-(D+2\Omega )\alpha_{1}-{\dot{\alpha }}_{1}+u+d_{f} \end{array}$$

In (10), the actual control u appears. Our target is to design u such that $e_{1},e_{2}$ converge to zero. Select a Lyapunov function V for the whole system as:

(11) $$V=\frac{1}{2}e_{1}{}^{T}e_{1}+\frac{1}{2}e_{2}{}^{T}e_{2}.$$

Its first time derivative is given by:

(12) $$\begin{array}{ll} \dot{V} & =e_{1}{}^{T}{\dot{e}}_{1}+e_{2}{}^{T}{\dot{e}}_{2}=e_{1}{}^{T}(X_{2}-{\dot{q}}_{d})+e_{2}{}^{T}{\dot{e}}_{2}\\ & =e_{1}{}^{T}(-c_{1}e_{1}+e_{2})+e_{2}{}^{T}[-(D+2\Omega )e_{2}-K(e_{1}+q_{d})-(D+2\Omega )\alpha_{1}-{\dot{\alpha }}_{1}+u+d_{f}] \end{array}.$$

We finally derive and design the real controller u. $\dot{V}$ must satisfy $\dot{V}\leq 0$. Some terms in (13) are definitely negative, and we shall keep them. Some terms are positive or indefinite, and we will use the control force to cancel them. Thus, we design the control effort as:

(13) $$u=-c_{2}e_{2}-e_{1}+(D+2\Omega )e_{2}+K(e_{1}+q_{d})+(D+2\Omega )\alpha_{1}+{\dot{\alpha }}_{1}-\rho \mathrm{sgn}(e_{2}),$$

where $c_{2}$ is a positive, definite, and symmetric matrix. The last term $-\rho \mathrm{sgn}(e_{2})$ in (15) is a robust compensator for the parameter variations and external disturbances.

Substituting Equation (13) into Equation (12) generates

(14) $$\dot{V}=-e_{1}{}^{T}c_{1}e_{1}-e_{2}{}^{T}c_{2}e_{2}+e_{2}{}^{T}d_{f}-\rho e_{2}{}^{T}\mathrm{sgn}(e_{2})\leq 0.$$

Because $-e_{1}{}^{T}c_{1}e_{1}\leq 0$, $-e_{2}{}^{T}c_{2}e_{2}\leq 0$, and $e_{2}{}^{T}d_{f}-\rho e_{2}{}^{T}\mathrm{sgn}(e_{2})\leq {\left\|e_{2}\right\|}_{1}{\left\|d_{f}\right\|}_{1}-\rho {\left\|e_{2}\right\|}_{1}\leq 0$, $\dot{V}$ coincides with zero if and only if the three terms are simultaneously equal to zero. Because of $c_{1}$ and $c_{2}$ being symmetric positive definite matrices, both $-e_{1}{}^{T}c_{1}e_{1}$ and $-e_{2}{}^{T}c_{2}e_{2}$ equal to zero if and only if $e_{1}=0$ and $e_{2}=0$. Therefore, $\dot{V}=0$ contains no trajectories other than ${\left[e_{1}^{T},e_{2}^{T}\right]}^{T}=0$. According to Lasalle’s invariance principle, the origin zero is globally asymptotically stable. Then, $e_{1},e_{2}\to 0$ as t→∞.

## 4. Adaptive Estimator

In the following, we will develop the procedure to deal with unknown system dynamics, lumped parametric uncertainties, and disturbances. The modified controller in (13) is

(15) $$u=-c_{2}e_{2}-e_{1}+\hat{D}(e_{2}+\alpha_{1})+\hat{K}(e_{1}+q_{d})+\hat{\Omega }(2e_{2}+2\alpha_{1})+{\dot{\alpha }}_{1}-\rho \mathrm{sgn}(e_{2}),$$

where $\hat{D},\hat{K}$ and $\hat{\Omega }$ are the estimates of D,K and Ω, respectively. Regarding the characteristics and performance of the proposed ABC strategy, we state the following theorem.

**Theorem** **1.** *In the presence of lumped disturbances $d_{f}$, the adaptive controller (15) with the adaptive estimator (16) applied to the microgyroscope model (3) guarantees that all the closed-loop signals are bounded and that state tracking errors converge to zero asymptotically.*

(16) $$\begin{array}{l} {\dot{\hat{D}}}^{T}=-\frac{1}{2}\gamma_{D}[(e_{2}+\alpha_{1})e_{2}{}^{T}+e_{2}(e_{2}+\alpha_{1}{)}^{T}]\\ {\dot{\hat{K}}}^{T}=-\frac{1}{2}\gamma_{K}[(e_{1}+q_{d})e_{2}{}^{T}+e_{2}(e_{1}+q_{d}{)}^{T}]\\ {\dot{\hat{\Omega }}}^{T}=\gamma_{\Omega }[e_{2}(e_{2}+\alpha_{1}{)}^{T}-(e_{2}+\alpha_{1})e_{2}{}^{T}] \end{array}$$

*where $\gamma_{D}>0,\gamma_{K}>0,\gamma_{\Omega }>0$.*

**Proof.**  Substituting (16) into (5) yields

(17) $$\{\begin{array}{c} {\dot{e}}_{1}=e_{2}+\alpha_{1}-{\dot{q}}_{d}\\ {\dot{e}}_{2}=[-c_{2}e_{2}-e_{1}+d_{f}-\rho \mathrm{sgn}(e_{2})]+\tilde{D}(e_{2}+\alpha_{1})+\tilde{K}(e_{1}+q_{d})+\tilde{\Omega }(2e_{2}+2\alpha_{1}) \end{array},$$

where $\tilde{D}=\hat{D}-D,\tilde{K}=\hat{K}-K,\tilde{\Omega }=\hat{\Omega }-\Omega$, represent the estimation errors.

Consider the Lyapunov function candidate as the form of (18):

(18) $$V=\frac{1}{2}e_{1}{}^{T}e_{1}+\frac{1}{2}e_{2}{}^{T}e_{2}+\frac{1}{2}\mathrm{tr}\{\gamma_{D}{}^{-1}\tilde{D}{\tilde{D}}^{T}\}+\frac{1}{2}\mathrm{tr}\{\gamma_{K}{}^{-1}\tilde{K}{\tilde{K}}^{T}\}+\frac{1}{2}\mathrm{tr}\{\gamma_{\Omega }{}^{-1}\tilde{\Omega }{\tilde{\Omega }}^{T}\},$$

where tr{⋅} is the matrix trace operator.

Differentiating (18) generates

(19) $$\begin{array}{ll} \dot{V} & =[-e_{1}{}^{T}c_{1}e_{1}-e_{2}{}^{T}c_{2}e_{2}+e_{2}{}^{T}d_{f}-\rho e_{2}{}^{T}\mathrm{sgn}(e_{2})]\\ & +e_{2}{}^{T}[\tilde{D}(e_{2}+\alpha_{1})+\tilde{K}(e_{1}+q_{d})+\tilde{\Omega }(2e_{2}+2\alpha_{1})]\\ & +\mathrm{tr}\{\gamma_{D}{}^{-1}\tilde{D}{\dot{\tilde{D}}}^{T}\}+\mathrm{tr}\{\gamma_{K}{}^{-1}\tilde{K}{\dot{\tilde{K}}}^{T}\}+\mathrm{tr}\{\gamma_{\Omega }{}^{-1}\tilde{\Omega }{\dot{\tilde{\Omega }}}^{T}\} \end{array}$$

Substituting the adaptive estimator (16) into (19), and $\dot{\hat{D}}={\dot{\hat{D}}}^{T},\dot{\hat{K}}={\dot{\hat{K}}}^{T},\dot{\hat{\Omega }}=-{\dot{\hat{\Omega }}}^{T}$, we obtain

(20) $$\dot{V}=-e_{1}{}^{T}c_{1}e_{1}-e_{2}{}^{T}c_{2}e_{2}+e_{2}{}^{T}d_{f}-\rho e_{2}{}^{T}\mathrm{sgn}(e_{2})\leq 0.$$

Note that (20) and (14) are identical. Thus, $e_{1}$ and $e_{2}$ converge to zero asymptotically. The adaptive laws that guarantee the tracking error converges to zero do not mean the parameter estimates are consistent only if the PE condition can be satisfied. Since the reference trajectories contain two distinct nonzero frequencies, the PE condition is satisfied, and the microgyroscope has sufficient persistence of excitation to permit the accurate identification of major fabrication imperfections and all the unknown system parameters. 

## 5. Simulation Study

The proposed ABC scheme was evaluated on a lumped *z*-axis microgyroscope sensor [1,2]. The physical parameters are described as:

$$\begin{array}{l} m=1.8\times 10^{-7}\mathrm{kg},k_{xx}=63.955\frac{N}{m},k_{yy}=95.92\frac{N}{m},k_{xy}=12.776\frac{N}{m}\\ d_{xx}=1.8\times 10^{-6}\frac{N\cdot s}{m},d_{yy}=1.8\times 10^{-6}\frac{N\cdot s}{m},d_{xy}=3.6\times 10^{-7}\frac{N\cdot s}{m} \end{array}$$

We chose 1 µm as the reference length $q_{0}$. It is known that the usual natural frequency of a microgyroscope is in the kHz range, so chose the $\omega_{0}$ as 1 kHz. Assume the unknown angular velocity is $\Omega_{z}=10\;\mathrm{rad}/s$. Non-dimensionalizing the physical parameters, we obtained the following nondimensional parameter matrices defined in (3):

$$D=\left[\begin{array}{cc} 0.01 & 0.002\\ 0.002 & 0.01 \end{array}\right],K=\left[\begin{array}{cc} 355.3 & 70.99\\ 70.99 & 532.9 \end{array}\right],\Omega =\left[\begin{array}{cc} 0 & -0.01\\ 0.01 & 0 \end{array}\right].$$

The desired trajectory should be the resonance of vibration modes. The reference trajectories were selected as $x_{d}=\cos(\omega_{1}t),y_{d}=\cos(\omega_{2}t)$, where *ω*_1_ = 6.17, *ω*_2_ = 5.11. Here $\omega_{1},\omega_{2}$ were chosen to be the resonance frequencies of the *z*-axis MEMS vibratory gyroscope. We assumed that $\omega_{1},\omega_{2}$ were fixed in the simulation period.

The lumped parametric uncertainties and external disturbances are given by $d_{f}=d-\Delta D\dot{q}-\Delta Kq$. As for model uncertainties, there were ±20% parameter variations for the spring and damping coefficients and ±20% magnitude changes in the coupling terms. Random signal $d=\left[\begin{array}{cc} randn(1,1) & randn(1,1) \end{array}\right]$ was considered as disturbance.

**Figure 3:**
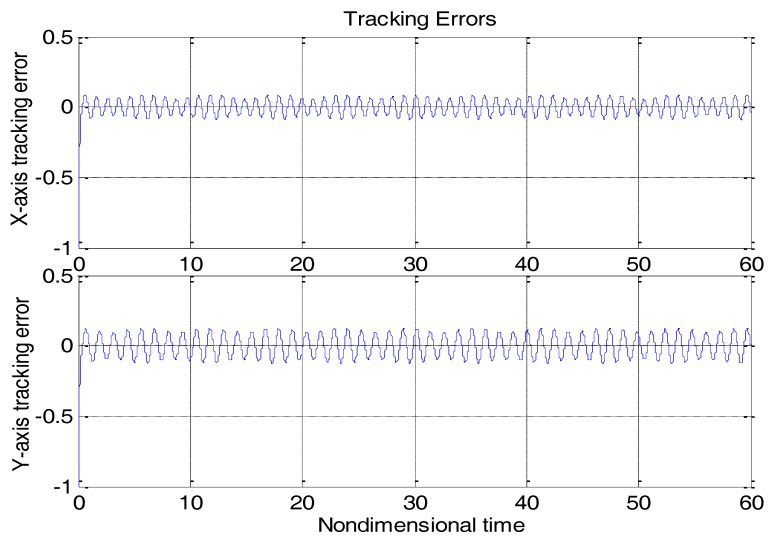
Tracking errors using a “dull” controller.

Let $D_{0},K_{0}$ and $\Omega_{0}$ to be the nominal values of D,K and Ω, respectively. Figure 3 shows the tracking error using a “dull” controller without any adaptation strategies by solely replacing D,K,Ω in (15) with $D_{0},K_{0},\Omega_{0}$. The control parameters are $c_{1}=c_{2}=20I$, where I is the unit matrix. For the moment, there is no disturbance. It must be noted that all of the system parameters, including the gyroscope, controller, and disturbance parameters are nondimensional herein, meaning that all of the parameters on vertical axes in the following figures are unitless. The simulation time was nondimensional, as were the simulation positions. Though they were nondimensional, the same class of parameters could be compared with each other, due to the unified reference physical quantity.

**Figure 4:**
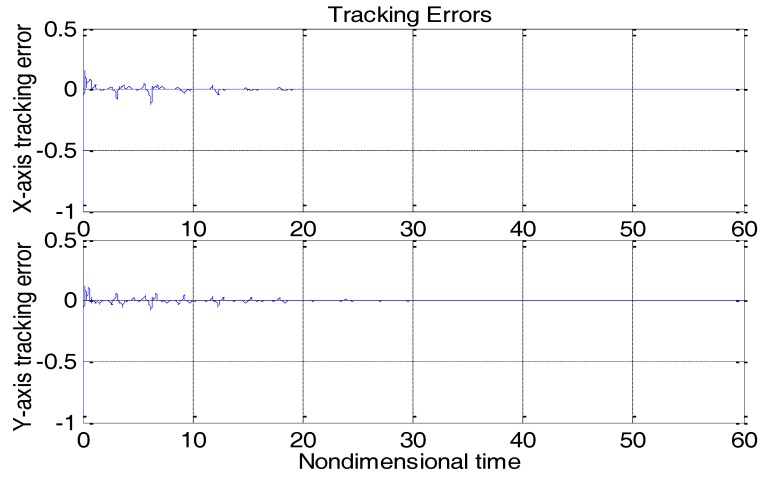
Tracking errors using the adaptive backstepping control (ABC) approach.

**Figure 5:**
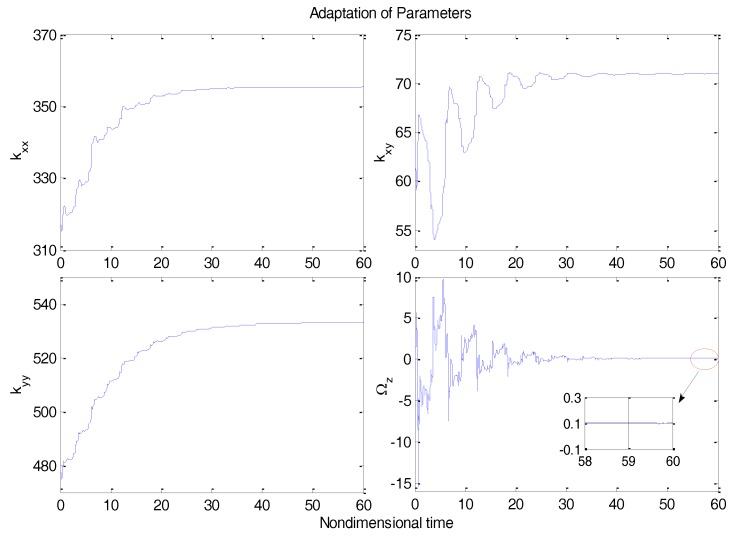
Adaptive parameter estimates using ABC.

**Figure 6:**
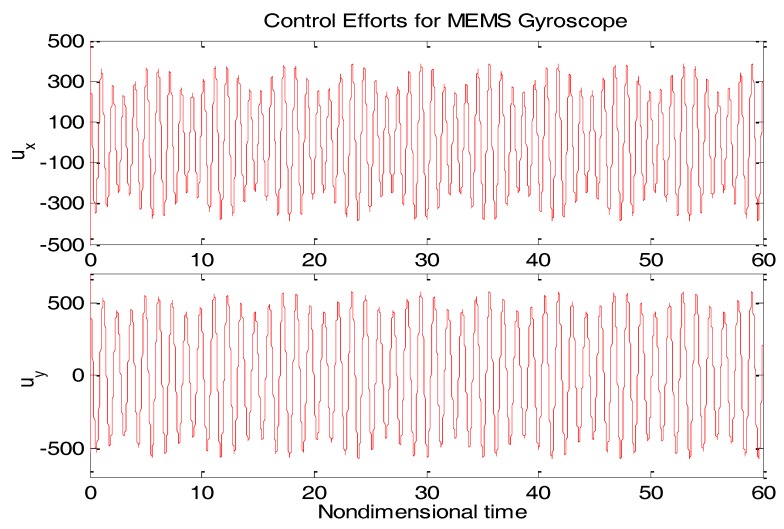
Control efforts for microgyroscope using ABC.

From Figure 3, due to the modeling error, the “dull” controller which relied on the nominal parameters led to a stable system, but the tracking errors were obvious. For comparison, Figure 4 depicts the tracking error using the proposed ABC approach, and Figure 5 shows the adaptation procedure of the parameter estimates. Figure 6 plots the control forces for the microgyroscope.

**Figure 7:**
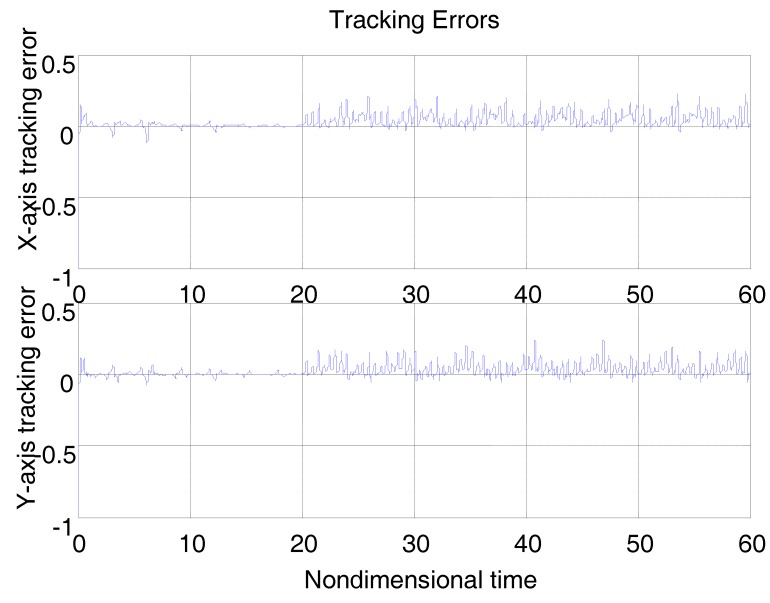
Tracking errors using ABC under step disturbances without robust term.

**Figure 8:**
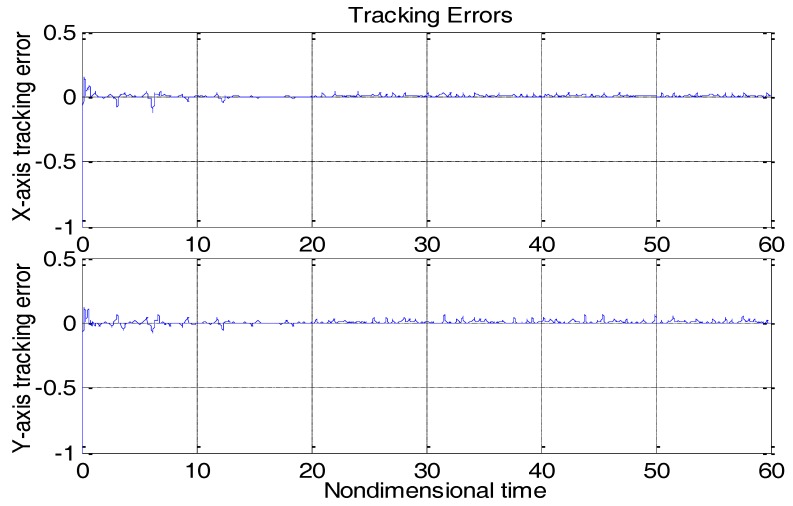
Tracking errors using ABC under step disturbances with robust term.

Obviously different from the result depicted in Figure 3, tracking errors approached zero quickly when using the proposed ABC scheme. Since the reference trajectories contained two different nonzero frequencies, the PE condition was satisfied. In Figure 5, the parameter estimates converged to their true values, including the angular velocity. Standard adaptive controllers are not always robust in the presence of model uncertainties and external disturbances. Hence, if $-\rho \mathrm{sgn}(e_{2})$ in (13) was relieved, our proposed control would not perform that well. For example, a step signal with an amplitude of 100 was added at 20 s as an external disturbance. Figure 7 shows the tracking errors using the adaptive controller without the robust term. Figure 8 exhibits the improvement of tracking errors using our proposed controller with the robust term $-\rho \mathrm{sgn}(e_{2})$. Comparing Figure 7 with Figure 8, the robust term effectively suppressed the disturbances and the tracking error maintained a very small value.

**Figure 9:**
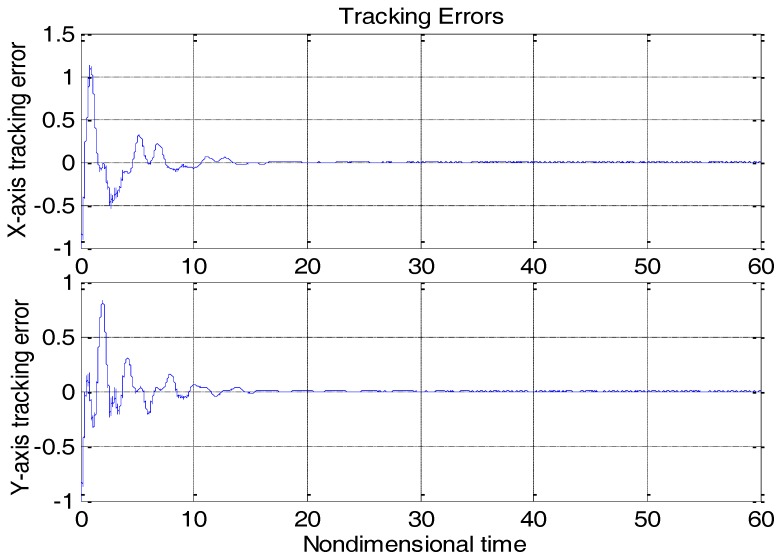
Tracking errors using the adaptive controller in [2].

**Figure 10:**
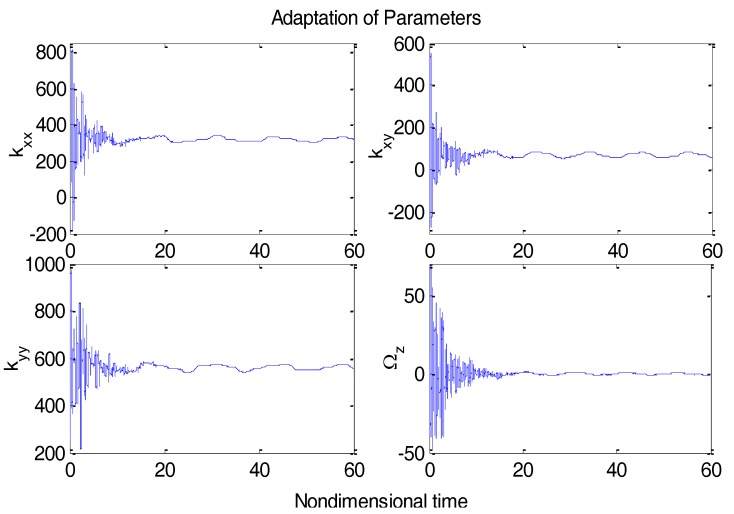
Adaptation of parameter estimates using the adaptive controller in [2].

**Figure 11:**
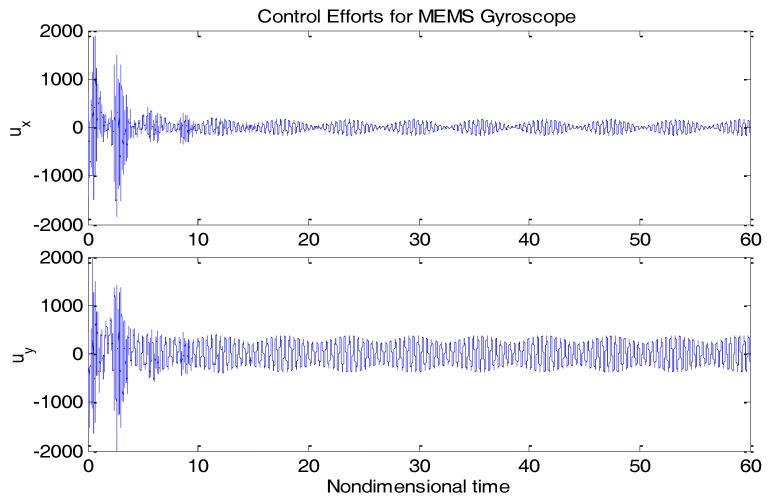
Control efforts for a microgyroscope using the adaptive controller in [2].

A well-known adaptive microgyroscope controller without the backstepping technique was presented in [2] by Park. The performance of our proposed ABC strategy was compared with the adaptive controller in [2]. Figure 9, Figure 10 and Figure 11 show the dynamic response using the adaptive controller in [2] with the same nominal gyroscope parameters under the same model uncertainties and disturbances.

The tracking errors with the adaptive controller displayed quite a large overshot at the beginning, as did the control efforts. The settling time of tracking errors was also worse than our proposed adaptive backstepping controller. The advantage of our proposed controller over the adaptive controller in the performance of parameter estimation is clear. Put simply, the proposed adaptive backstepping controller could improve the dynamic and static performance of the microgyroscope.

## 6. Conclusions

An adaptive control with backstepping technique for a *z*-axis microgyroscope was investigated and analyzed. The dynamics model of the microgyroscope was developed and transformed to aid in the backstepping control design. A backstepping approach and adaptive strategy were utilized to deal with the model uncertainties, disturbances, and unknown parameters of the microgyroscope. A controller was designed to recursively and progressively step back out of the subsystem, guaranteeing stability at each step until reaching the final external control step. Consistent parameter estimates, asymptotic stability, and tracking performance under the lumped disturbances were proved based on a Lyapunov analysis. Numerical simulation examples demonstrated the validity of the proposed ABC scheme, showing the improved performance and consistent parameter estimation. 

In our study, we only emphasized the proposed adaptive backstepping control algorithm on the microgyroscope model. In the next step, the proposed adaptive backstepping controller should be implemented in a practical experimental system to verify its effectiveness.
